# Supplementary material for: Self-compliant ionic skin by leveraging hierarchical hydrogen bond association
Source: Nat Commun. 2024 Jan 30;15:885. doi: 10.1038/s41467-024-45079-4 (PMC10825218; doi:10.1038/s41467-024-45079-4)
Supplement: Supplementary file 1 — Supplementary Information [file 41467_2024_45079_MOESM1_ESM.pdf]

Supplementary Information

Self-compliant ionic skin by leveraging hierarchical hydrogen bond association

Huating Ye[1], Baohu Wu[2], Shengtong Sun[1,*], and Peiyi Wu[1,*]

[1]State Key Laboratory for Modification of Chemical Fibers and Polymer Materials, College of Chemistry and Chemical Engineering & Center for Advanced Low-dimension Materials, Donghua University, Shanghai 201620, China

[2]Jülich Centre for Neutron Science (JCNS) at Heinz Maier-Leibnitz Zentrum (MLZ) Forschungszentrum Jülich, Lichtenbergstr. 1, 85748 Garching, Germany

Correspondence should be addressed to S.T.S. (email: shengtongsun@dhu.edu.cn) and P.Y.W. (email: wupeiyi@dhu.edu.cn)

This file includes

Suppl. Fig. 1. Transmittance spectra of P(BA-*co*-MAA) ionogels

Suppl. Fig. 2. DSC heating curves of P(BA-*co*-MAA) ionogels

Suppl. Fig. 3. Tensile stress-strain curve of P(BA-*co*-$MAA_{0.04}$) ionogel

Suppl. Fig. 4. Tensile stress-strain curves of P(BA-*co*-$MAA_{0.3\ to\ 1}$) ionogels

Suppl. Fig. 5. Rheological curves of P(BA-*co*-MAA) ionogels

Suppl. Fig. 6. Cyclic tensile stress-strain curves of P(BA-*co*-$MAA_{0.05}$) ionogel

Suppl. Fig. 7. Rheological curves of P(BA-*co*-$MAA_{0.05}$) ionogel at different temperatures

Suppl. Fig. 8. Temperature-sweep rheological curve of P(BA-*co*-$MAA_{0.05}$) ionogel

Suppl. Fig. 9. Apparent association energy of P(BA-*co*-$MAA_{0.05}$) ionogel

Suppl. Fig. 10. Rheological curves and association energies of ionogels with different compositions

Suppl. Fig. 11. Rheological curves and strain relaxation of P(BA-*co*-$MAA_{0.05}$) elastomer

Suppl. Fig. 12. Rheology and association energies of P(BA-*co*-AA) and P(BA-*co*-MAANa) ionogels

Suppl. Fig. 13. Deconvoluted IR analysis of P(BA-*co*-$MAA_{0.05}$) ionogel

Suppl. Fig. 14. 2D low-field ^{1}H NMR spectra of P(BA-*co*-$MAA_{0.05}$) ionogel

Suppl. Fig. 15. GPC profile of as-prepared P(BA-*co*-$MAA_{0.05}$)

Suppl. Fig. 16. Tensile and rheological behavior of recast P(BA-*co*-$MAA_{0.05}$) ionogel

Suppl. Fig. 17. Tensile curves of P(BA-*co*-$MAA_{0.05}$) ionogel before and after immersing in water

Suppl. Fig. 18. Spontaneous penetration of adhesive materials into microstructured PET substrate

Suppl. Fig. 19. Dynamic compliance of adhesive materials on repeatedly inflated/deflated balloon

Suppl. Fig. 20. Cell viability test of L929 cells incubated with different amounts of ionogel

Suppl. Fig. 21. On-skin irritation test of P(BA-*co*-MAA) ionogel

Suppl. Fig. 22. SEM images of liquid metal layer and particle distribution

Suppl. Fig. 23. Stress relaxation of P(BA-*co*-MAA) ionogel electrodes with/without liquid metal

Suppl. Fig. 24. Rheological curves of P(BA-*co*-MAA) ionogel electrodes with/without liquid metal

Suppl. Fig. 25. SNRs of ECG signals recorded by P(BA-*co*-MAA) ionogel and commercial gel electrodes

Suppl. Table 1. Comparison of the frequency ranges for the gel point state among different materials

Suppl. Table 2. Signs of the main cross-peaks in 2DCOS synchronous and asynchronous spectra

References S1-S5

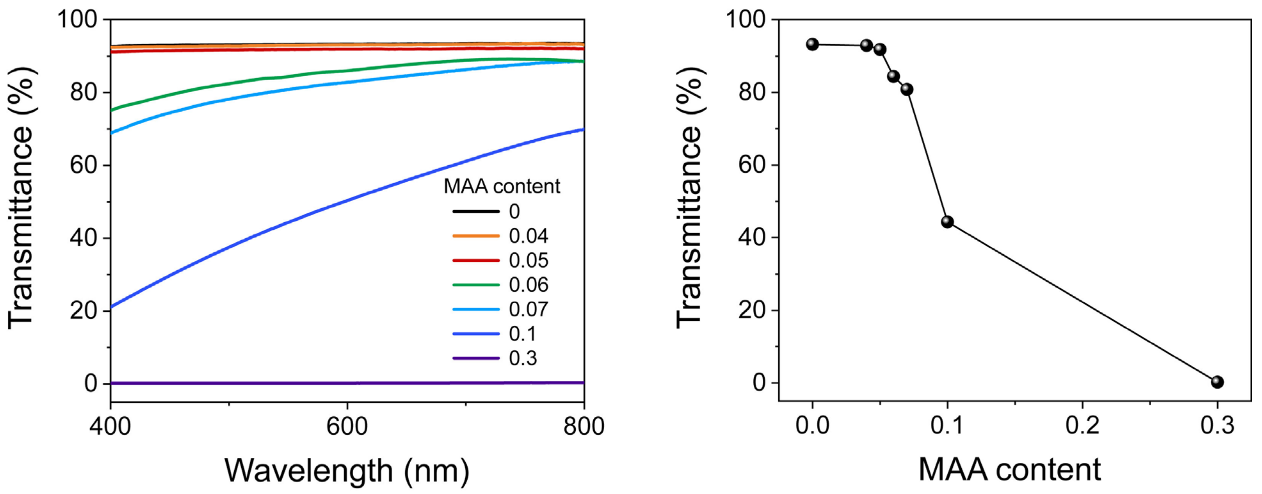

Supplementary Fig. 1. Transmittance spectra and changes of P(BA-*co*-MAA) ionogels with increasing MAA contents. The thicknesses of all the measured samples were fixed to 500 µm. The transmittance of the optimized P(BA-*co*-$MAA_{0.05}$) ionogel is as high as 92%. Dramatic transmittance reduction took place at the MAA contents of 0.1 and 0.3 due to the formation of large PMAA-rich H-bond aggregates that strongly scatter incident visible light.

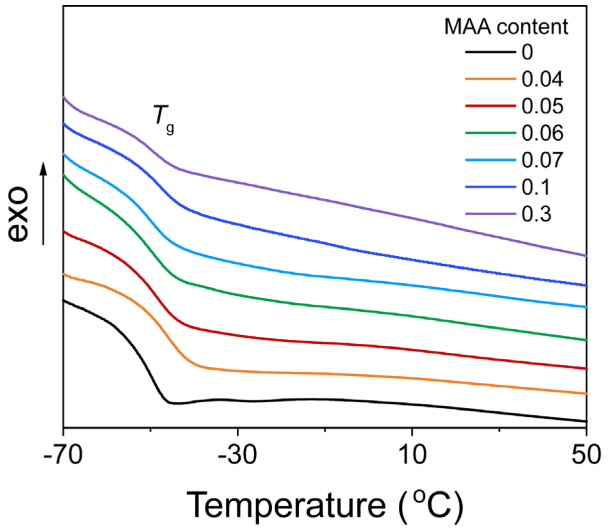

Supplementary Fig. 2. DSC heating curves of P(BA-*co*-MAA) ionogels with increasing MAA contents. No significant changes of glass transition temperatures (T_gs) were observed with varying MAA molar contents. This is probably due to the incompatibility of PMAA moieties with PBA and ionic liquid, which did not remarkably affect the segmental mobility of PBA-rich matrix at the studied compositions.

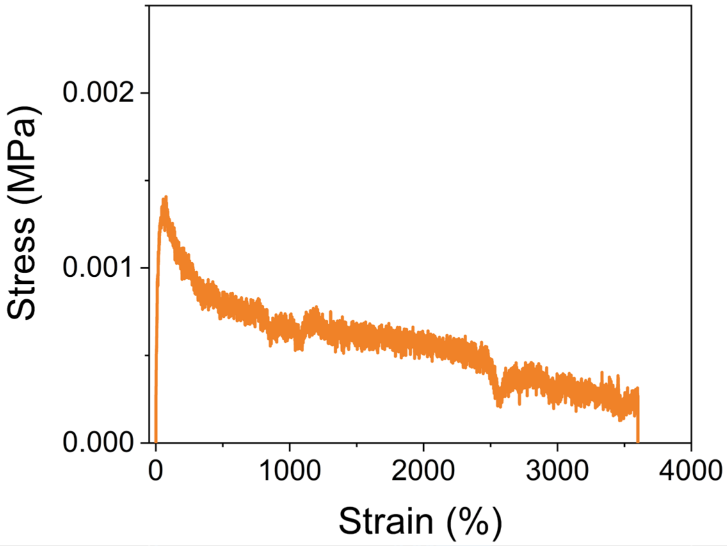

Supplementary Fig. 3. Tensile stress-strain curve of P(BA-*co*-$MAA_{0.04}$) ionogel. Strain-softening behavior was observed for this sample due to its viscous nature.

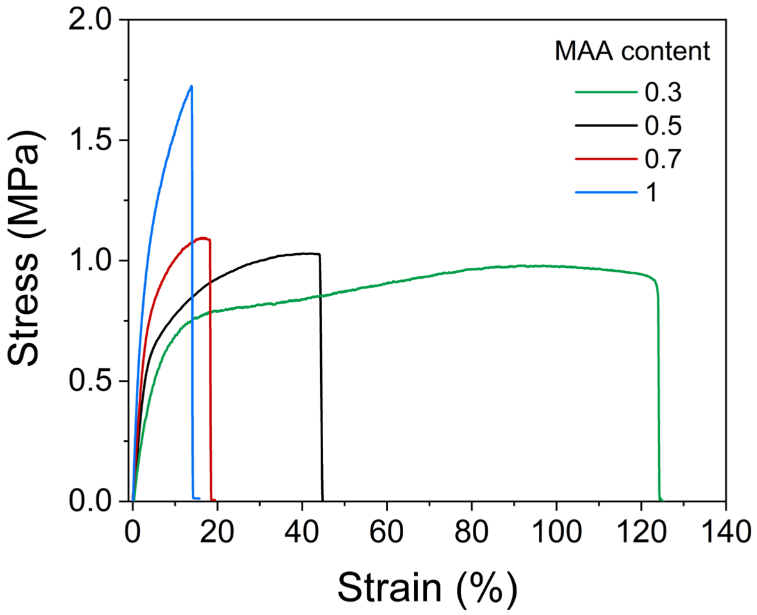

Supplementary Fig. 4. Tensile stress-strain curves of P(BA-*co*-MAA) ionogels with the MAA molar contents from 0.3 to 1. With increasing MAA contents to be larger than 0.3, the ionogels became even stiffer with significantly reduced stretchability.

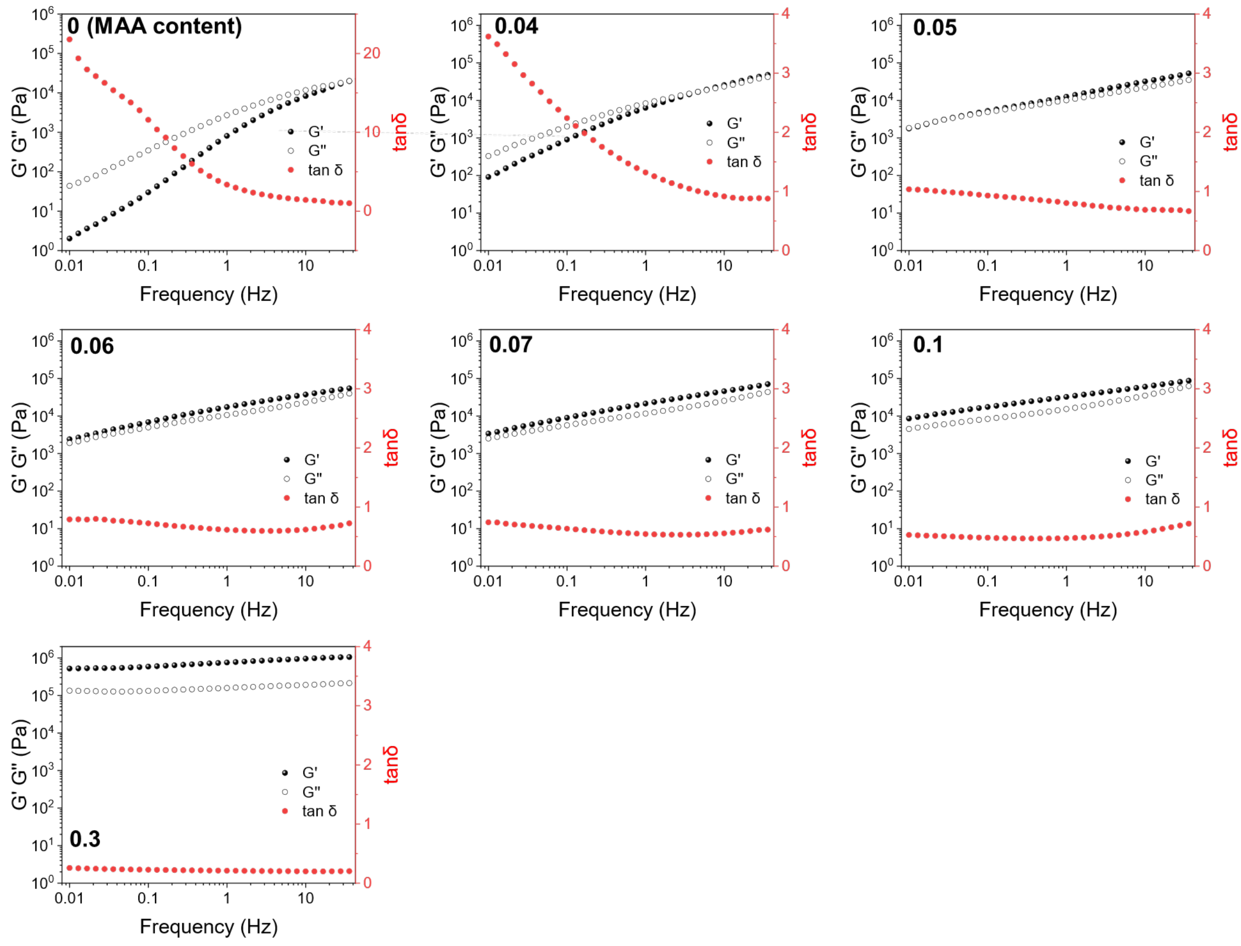

Supplementary Fig. 5. Frequency-sweep rheological curves of P(BA-*co*-MAA) ionogels with increasing MAA contents. The tan δ value at 0.1 Hz was chosen as the evaluating factor for Figure 2g.

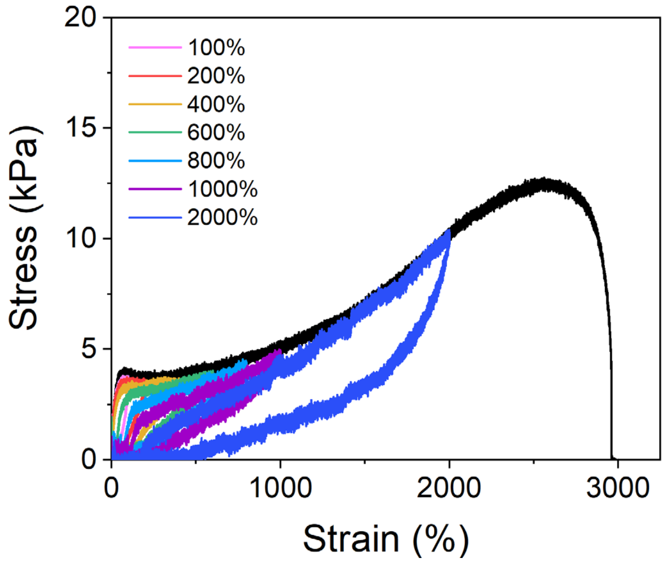

Supplementary Fig. 6. Cyclic tensile stress-strain curves of P(BA-*co*-MAA$_{0.05}$) ionogel with increasing strains. All the cyclic tensile curves show good strain recovery and coincide well with the single curve to break, suggesting the good elasticity of the ionogel.

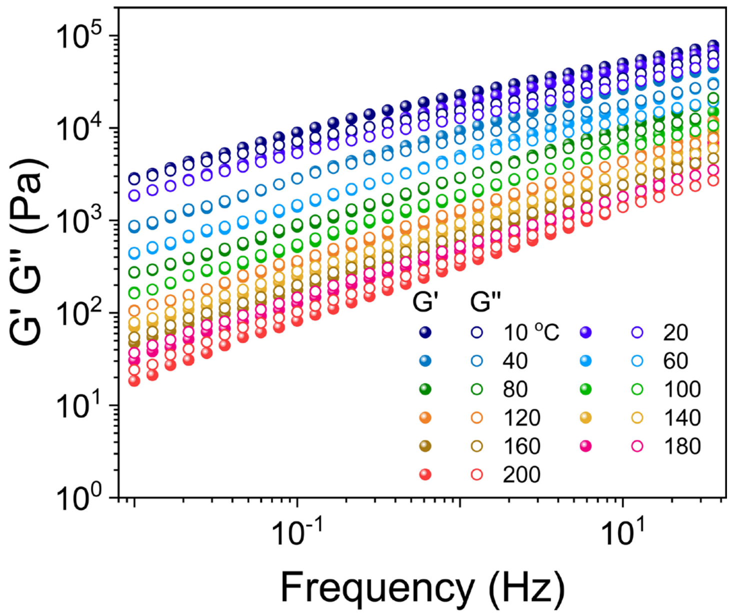

Supplementary Fig. 7. Frequency-sweep rheological curves of P(BA-*co*-MAA$_{0.05}$) ionogel at different temperatures.

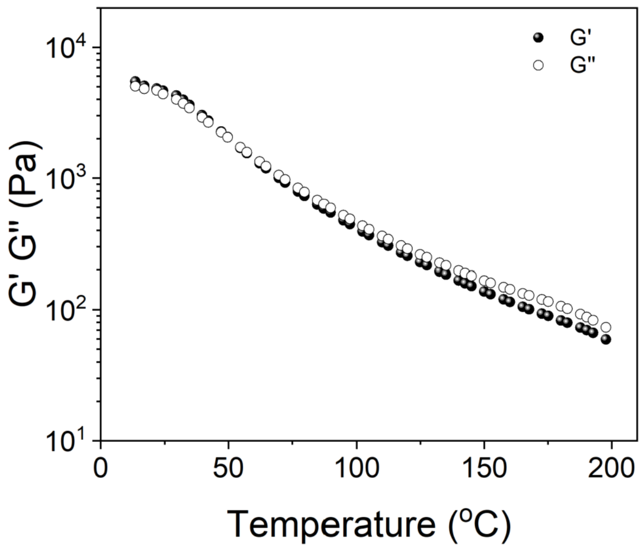

Supplementary Fig. 8. Temperature-sweep rheological curve of P(BA-*co*-MAA$_{0.05}$) ionogel at 0.1 Hz. The *G*' and *G*'' curves overlapped in a wide temperature range, consolidating the presence of gel point state almost independent of frequency and temperature changes.

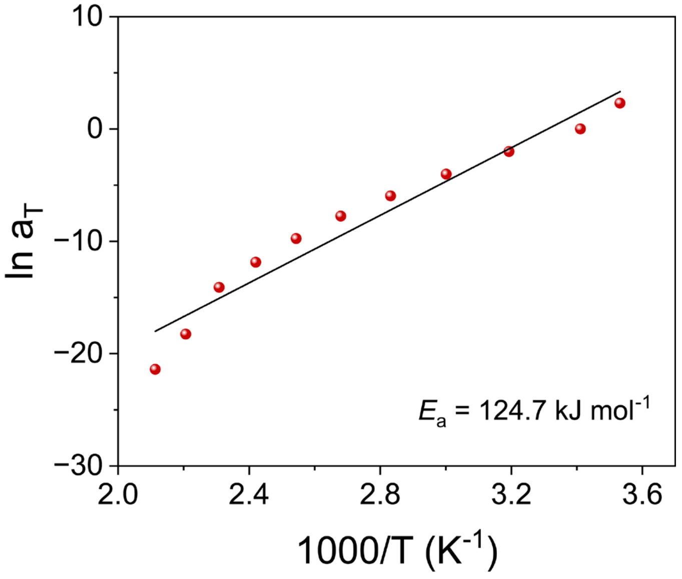

Supplementary Fig. 9. Calculation of the apparent association energy of P(BA-*co*-MAA$_{0.05}$) ionogel. The activation energy was calculated from the Arrhenius equation $a_T = Ae^{-Ea/RT}$, where a_T is the horizontal shift factor in the time-temperature superposition rheological curves (Fig. 2h), A the pre-exponential factor, E_a the activation energy, R the ideal gas constant, and T the Kelvin temperature.

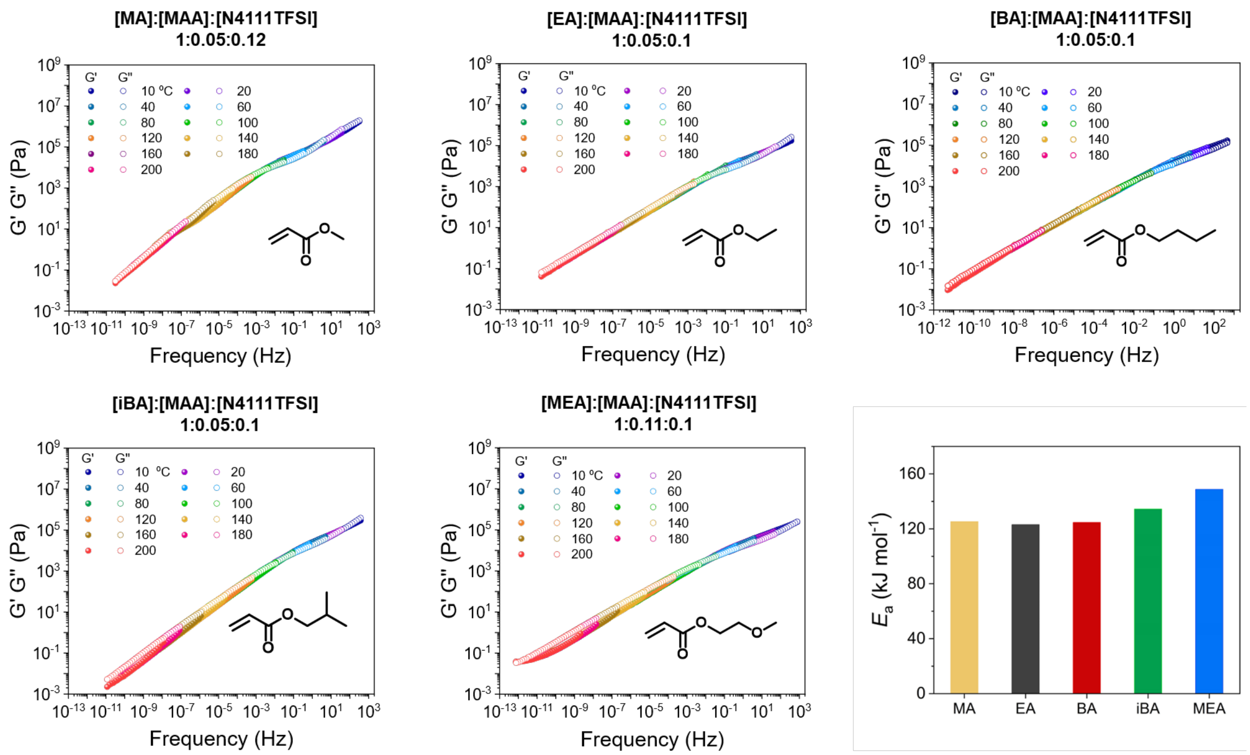

Supplementary Fig. 10. Rheological master curves and corresponding association energies of P(MA-*co*-MAA), P(EA-*co*-MAA), P(BA-*co*-MAA), P(iBA-*co*-MAA), and P(MEA-*co*-MAA) ionogels. The optimized ionogels all showed the gel point state at a super-wide frequency range. The calculated association energies in the cases of P(MA-*co*-MAA), P(EA-*co*-MAA), and P(BA-*co*-MAA) are very approximate to each other, demonstrating the dominating role of PMAA-related H-bond associations. Slightly higher association energies in the case of P(iBA-*co*-MAA) and P(MEA-*co*-MAA) ionogels may arise from the increased steric hindrance and ether-carboxylic acid H-bond in the side groups, respectively.

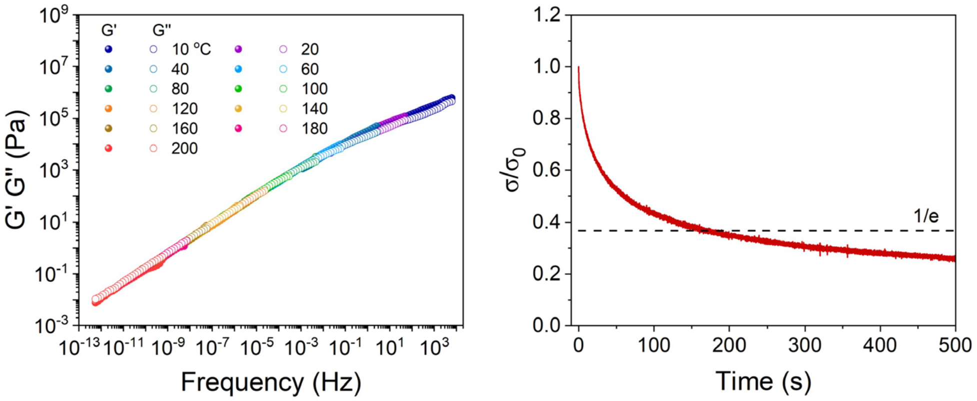

Supplementary Fig. 11. Rheological master curves and strain relaxation behavior of P(BA-*co*-MAA$_{0.05}$) elastomer. Similar to the ionogel, P(BA-*co*-MAA$_{0.05}$) elastomer exhibited also the gel point state in a super-wide frequency range, as well as good stress relaxation behavior (relaxation time: 166 s).

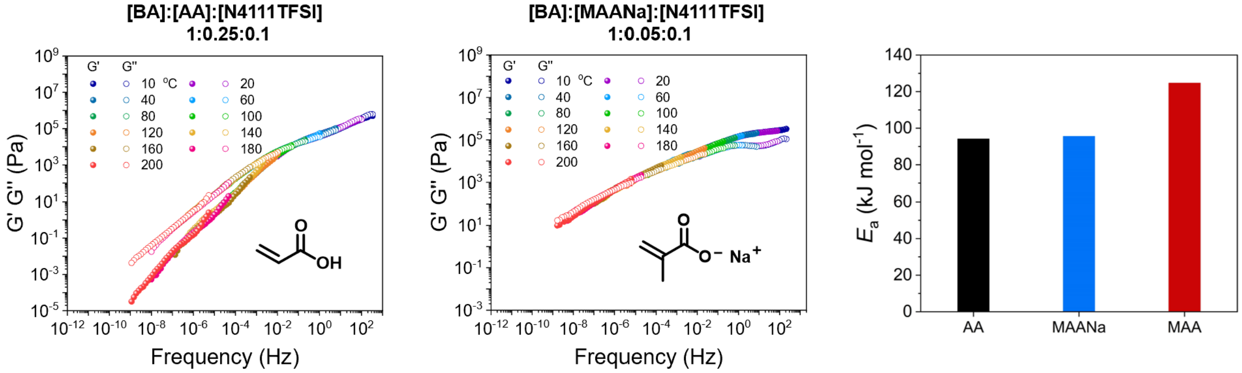

Supplementary Fig. 12. Rheological master curves and corresponding association energies of P(BA-*co*-AA) and P(BA-*co*-MAANa) ionogels. For comparison, the compositions of the two ionogels were first optimized to reach the gel point state at 20 °C. It is noted that the gel point states of these two ionogels are very short. This is probably due to the weakened binding strength that could not maintain stable association at lower frequencies, as evidenced by their lower association energies than P(BA-*co*-MAA$_{0.05}$) ionogel.

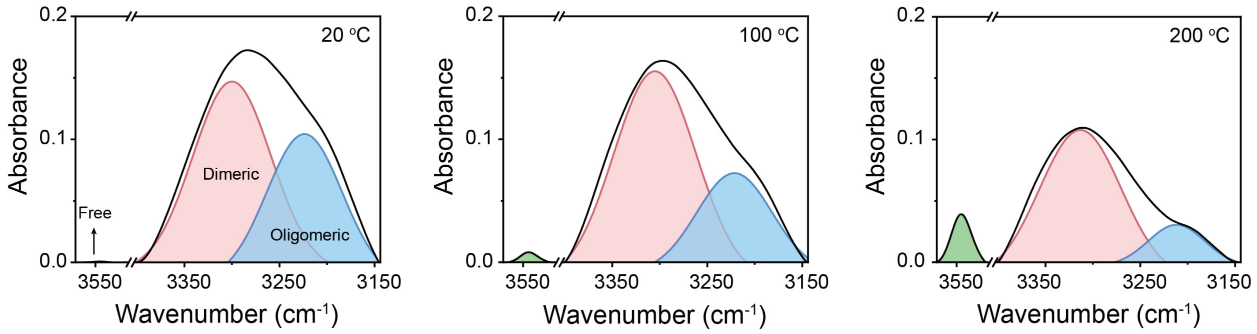

Supplementary Fig. 13. Deconvoluted IR analysis of P(BA-*co*-MAA$_{0.05}$) ionogel at 20, 100, and 200 °C. The transformation from H-bonded COOH to free COOH can be clearly evidenced by the peak area changes of corresponding peaks.

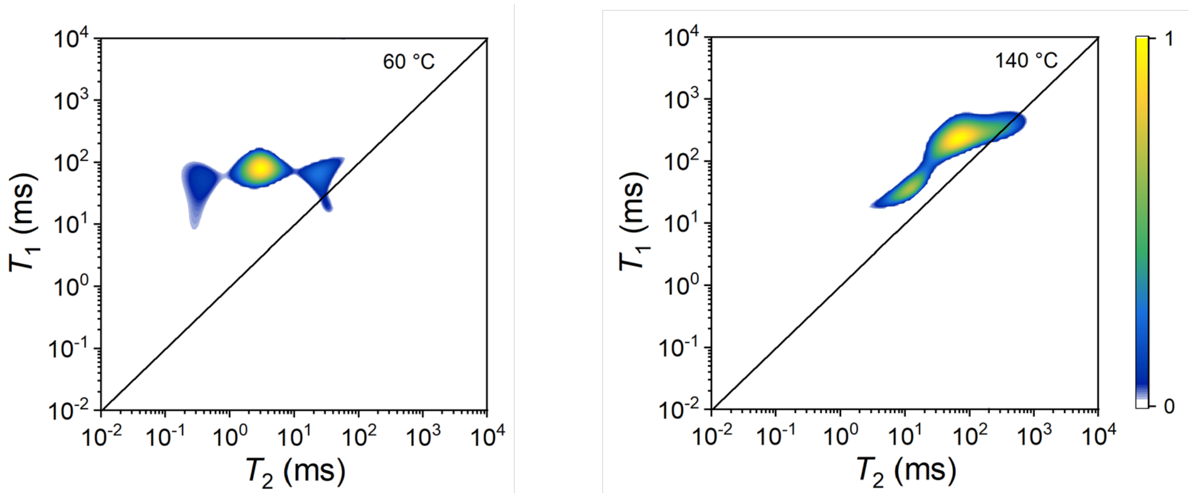

Supplementary Fig. 14. 2D low-field ^{1}H NMR spectra of P(BA-*co*-MAA$_{0.05}$) ionogel at 60 and 140 °C, respectively.

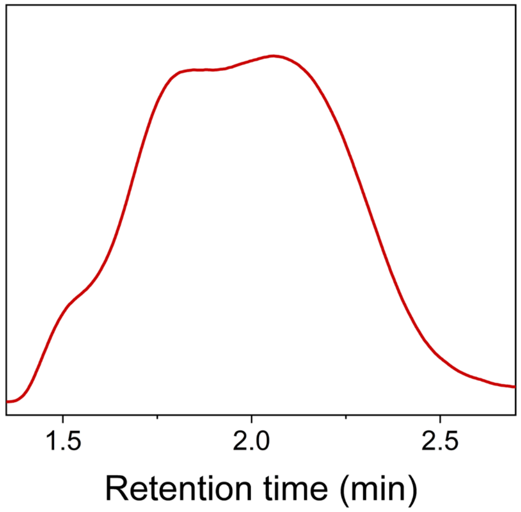

Supplementary Fig. 15. GPC profile of as-prepared P(BA-*co*-MAA$_{0.05}$). The copolymer has a number-average molecular weight of 1.1×10^5 g mol^{-1} with a polydispersity index (PDI) of 1.3.

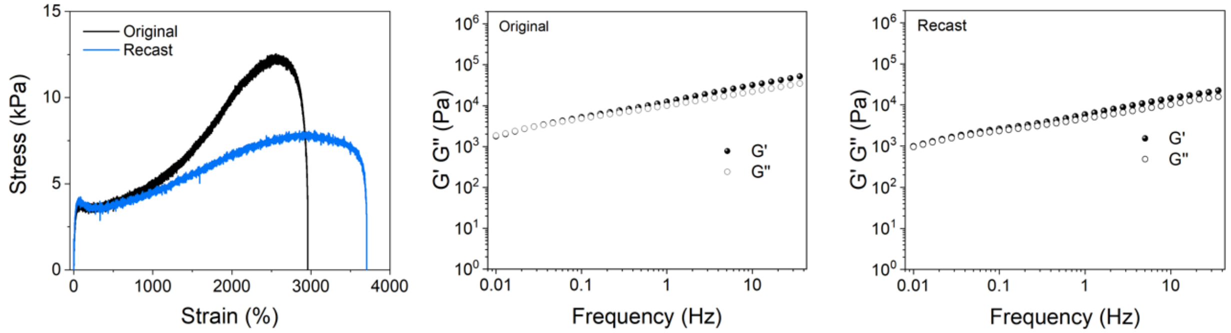

Supplementary Fig. 16. Tensile and rheological behavior of recast P(BA-*co*-MAA$_{0.05}$) ionogel. Both the mechanical properties and gel-point-state behavior can be reproduced by recasting the ionogel from its solution in ethyl acetate.

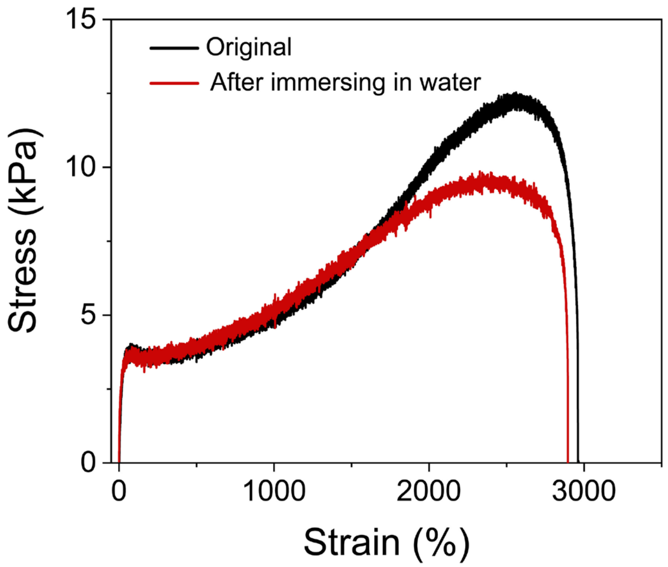

Supplementary Fig. 17. Tensile curves of P(BA-*co*-MAA$_{0.05}$) ionogel before and after immersing in water for 12 h.

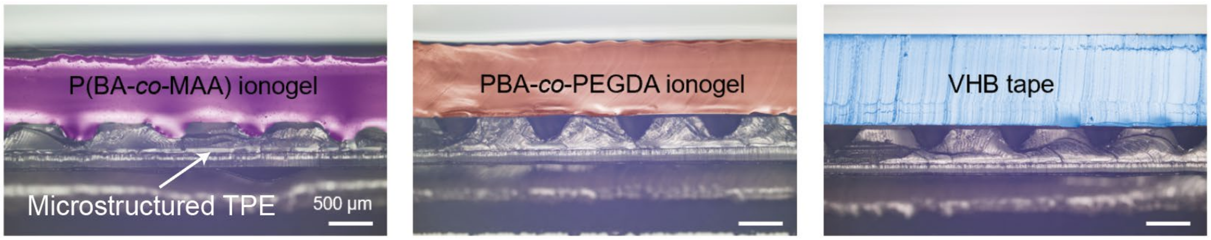

Supplementary Fig. 18. Spontaneous penetration of three adhesive materials into the microstructure of PET substrate. Only P(BA-*co*-MAA) ionogel at the gel point state can spontaneously penetrate the microstructure of the substrate, while apparent gaps were observed in the cases of elastic PBA-*co*-PEGDA ionogel and VHB tape.

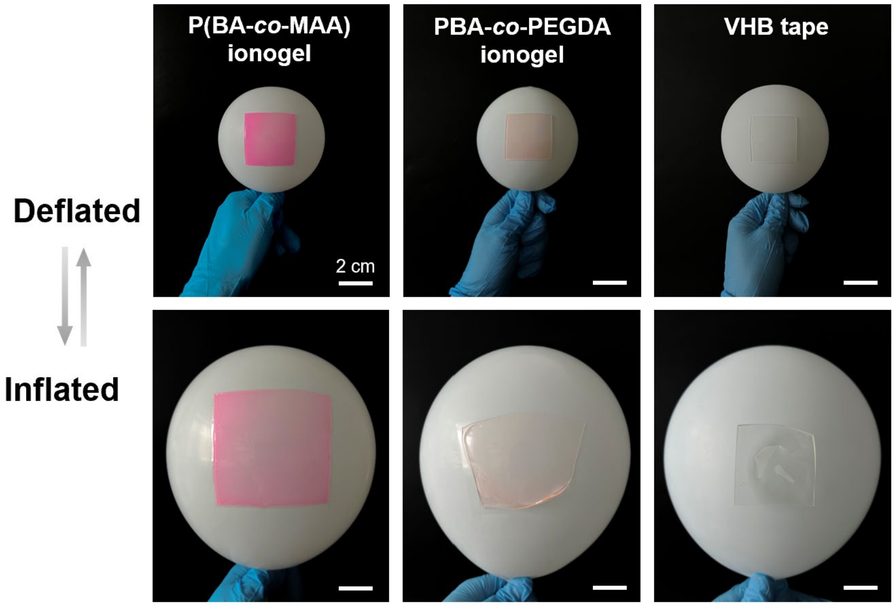

Supplementary Fig. 19. Dynamic compliance of three adhesive materials on a repeatedly inflated/deflated balloon. The size of samples is 3×3 cm^2. After 200 cycles of inflation/deflation, the self-compliant P(BA-*co*-MAA) ionogel remained conformal with the balloon, while elastic PBA-*co*-PEGDA ionogel and VHB tapes all failed with interfacial fracture.

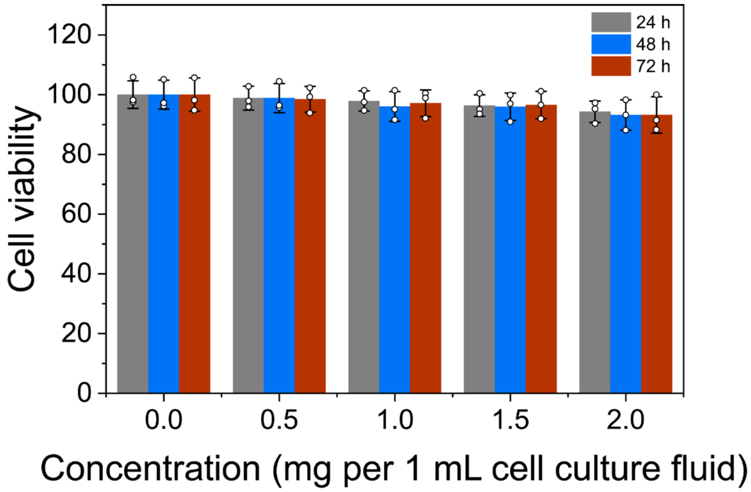

Supplementary Fig. 20. Cell viability test of L929 cells incubated with different amounts of P(BA-*co*-MAA) ionogel for 72 h. The ionogel has excellent biocompatibility with cell viabilities larger than 93%. Data are presented as the mean values ± SD, n = 3 independent samples. Source data are provided as a Source Data file.

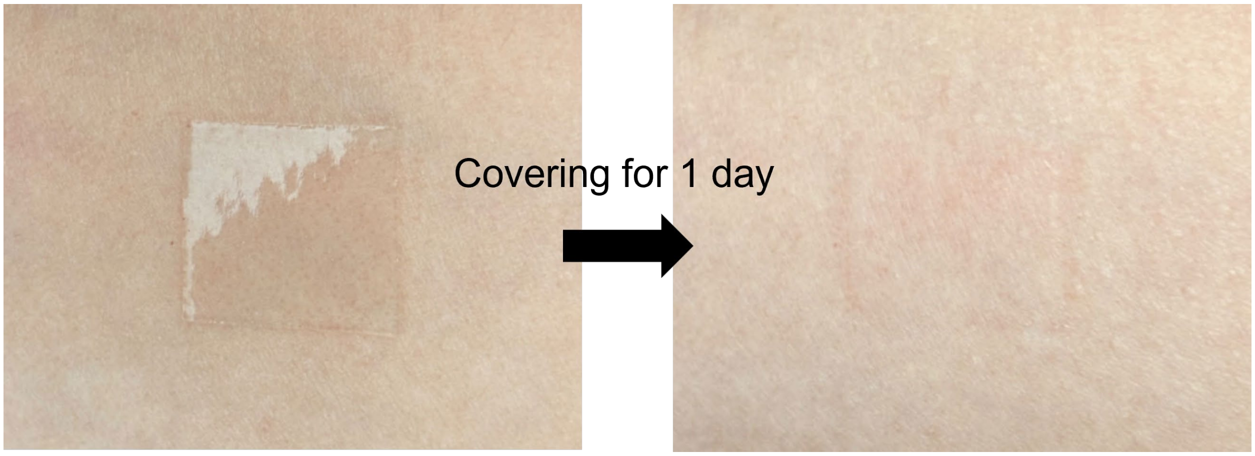

Supplementary Fig. 21. On-skin irritation test of P(BA-*co*-MAA) ionogel. The ionogel was attached on the left forearm for one day. There was no obvious irritation or injury to human skin, suggesting that the ionogel is skin-friendly.

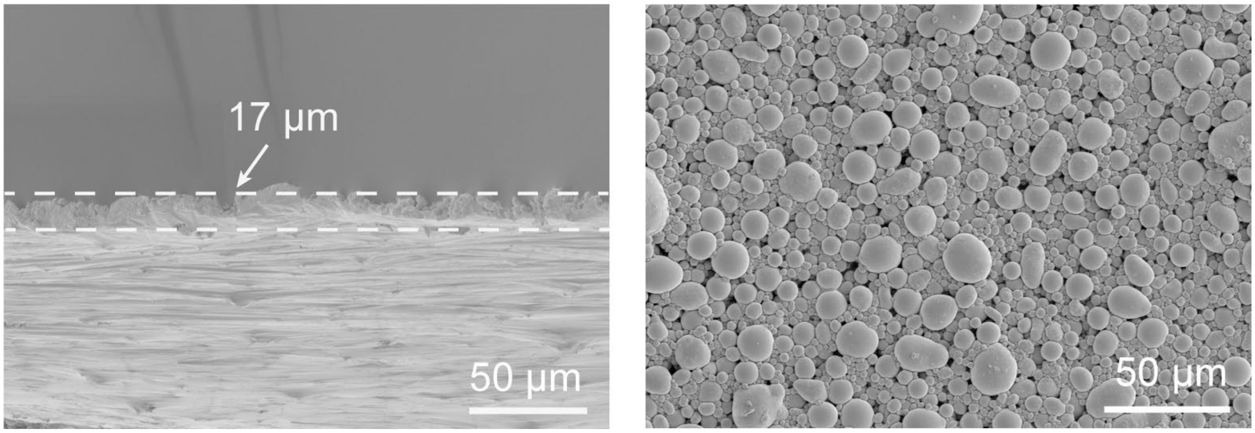

Supplementary Fig. 22. SEM images of liquid metal layer and particle distribution. The thickness of the liquid metal layer was ~17 µm, and the liquid metal particle size was 3-15 µm.

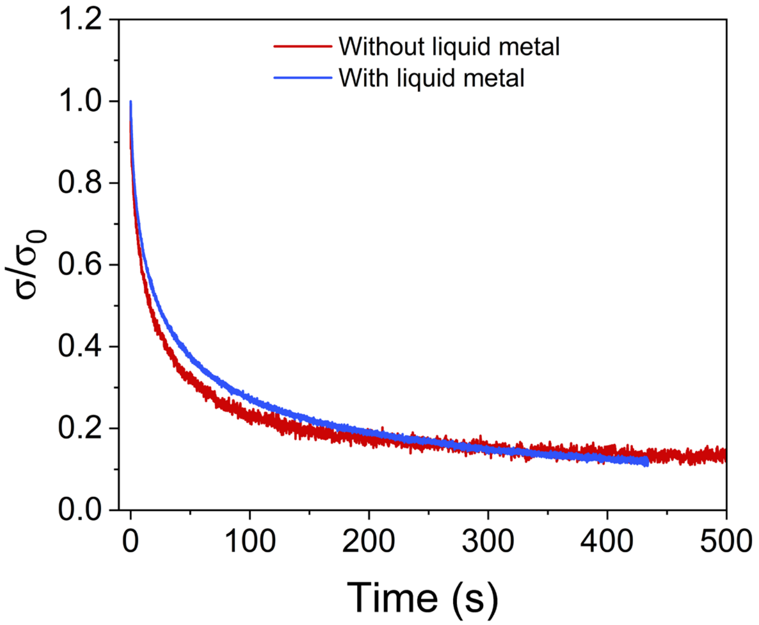

Supplementary Fig. 23. Stress relaxation curves of P(BA-*co*-MAA) ionogel electrodes with/without liquid metal. The introduction of liquid metal did not significantly affect the stress relaxation behavior of P(BA-*co*-MAA) ionogel.

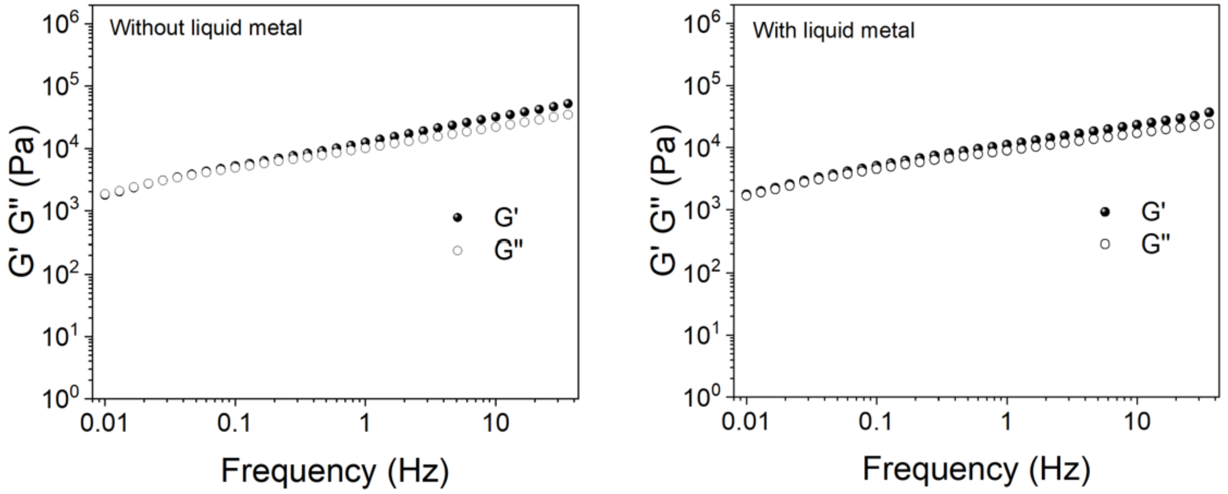

Supplementary Fig. 24. Rheological curves of P(BA-*co*-MAA) ionogel electrodes with/without liquid metal. The rheological data were obtained at the fixed oscillation strain of 0.1% and 20 ºC.

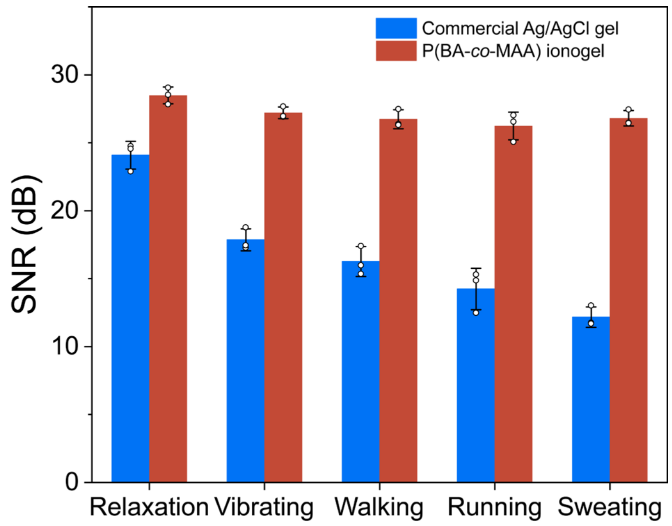

Supplementary Fig. 25. SNRs of ECG signals recorded by P(BA-*co*-MAA) ionogel and commercial gel electrodes under the states of relaxation, vibrating, walking, running, and sweating. Data are presented as the mean values ± SD, n = 3 independent samples. Source data are provided as a Source Data file.

Supplementary Table 1. Comparison of the frequency ranges for the gel point state among different materials.

**Gel-point-state polymers**	**Frequency range (Hz)**	**Span of orders of magnitude**	**Refs.**
P(BA-*co*-MAA) ionogel	10^{-11} – 500	13	This work
Gel-point adhesive patch	0.1 – 10	2	[S1]
PMEA-PATU	10^{-5} – 10^{3}	8	[S2]
p($BA_{2.8}$-$AA_{1.0}$)	0.016 – 16	3	[S3]
ACC-PAA hydrogel	10 – 10^{5}	4	[S4]
ACC/PAA/alginate hydrogel	0.1 – 70	2	[S5]

Supplementary Table 2. Signs of the main cross-peaks in 2DCOS synchronous and asynchronous spectra.

3220	-	-	-	-	
3240	-	-	-		
3330	+	-			
3342	+				
3545					
	3545	3342	3330	3240	3220

2DCOS, as a mathematical method, is very suitable for the investigation of the variations of chemical groups. Its basic principle was first proposed by Noda and has been applied more and more widely to follow spectral variations under various external perturbations, such as time, temperature, pressure, concentration, and other physical variables. By spreading the original spectral information along a second dimension, spectral resolution enhancement can be achieved, allowing the additional important information about molecular motions or conformational changes not readily visible in conventional analysis to be extracted.

2DCOS includes two types of correlation maps, synchronous and asynchronous spectra. The judging rule of the sequence can be summarized as Noda's rule- that is, if the multiplication of the signs of cross-peaks (ν_1, ν_2, and assume $\nu_1 > \nu_2$) in synchronous and asynchronous spectra is positive, the change at ν_1 may occur prior to ν_2, and vice versa. In Supplementary Table 2, "+" means the same signs in synchronous and asynchronous spectra, while "-" means different signs in synchronous and asynchronous spectra. Judging from the above rule, the sequential order is 3220 cm^{-1} → 3240 cm^{-1} → 3545 cm^{-1} → 3330 cm^{-1} → 3342 cm^{-1} (→ means prior to or earlier than), i.e. ν(O-H) (oligomeric H-bond) (strong) → ν(O-H) (oligomeric H-bond) (weak) → ν(O-H) (free COOH) → ν(O-H) (dimeric H-bond) (strong) → ν(O-H) (dimeric H-bond) (weak).

Supplementary references

S1. Lin X. *et al.* A viscoelastic adhesive epicardial patch for treating myocardial infarction. *Nat. Biomed. Eng.* **3**, 632-643 (2019).

S2. Wang, Y.-J. *et al.* Polymer pressure-sensitive adhesive with a temperature-insensitive loss factor operating under water and oil. *Adv. Funct. Mater.* **31**, 2104296 (2021).

S3. Wu, J., Wu, B., Xiong, J., Sun, S. & Wu, P. Entropy-mediated polymer–cluster interactions enable dramatic thermal stiffening hydrogels for mechanoadaptive smart fabrics. *Angew. Chem. Int. Ed.* **61**, e202204960 (2022).

S4. Niu, W., Zhu, J., Zhang, W. & Liu, X. Simply formulated dry pressure-sensitive adhesives for substrate-independent underwater adhesion. *ACS Mater. Lett.* **4**, 410-417 (2022).

S5. Lei, Z., Wang, Q., Sun, S., Zhu, W. & Wu, P. A bioinspired mineral hydrogel as a self-healable, mechanically adaptable ionic skin for highly sensitive pressure sensing. *Adv. Mater.* **29**, 1700321 (2017).
