# Supplementary material for: Self-compliant ionic skin by leveraging hierarchical hydrogen bond association
Source: Nat Commun. 2024 Jan 30;15:885. doi: 10.1038/s41467-024-45079-4 (PMC10825218; doi:10.1038/s41467-024-45079-4)
Supplement: Supplementary file 3 — Description of Additional Supplementary Files [file 41467_2024_45079_MOESM3_ESM.pdf]

Description of Additional Supplementary Files

File Name: Supplementary Movie 1
Description: Flaw insensitivity of PBA-co-MAA ionogel.

File Name: Supplementary Movie 2
Description: Comparison of dynamic compliance with stretchable substrate.

File Name: Supplementary Movie 3
Description: Comparison of stress relaxation behavior upon steel ball impact.
